# Pre-hospital management of traumatic cardiac arrest 2024 position statement: Faculty of Prehospital Care, Royal College of Surgeons of Edinburgh

**DOI:** 10.1186/s13049-024-01304-z

**Published:** 2024-12-31

**Authors:** Celestine Weegenaar, Zane Perkins, David Lockey

**Affiliations:** Faculty of Pre-Hospital Care, Royal College of Surgeons of Edinburgh, Edinburgh, UK

**Keywords:** Traumatic cardiac arrest, Pre-hospital, Cardiac arrest in trauma, Out of hospital cardiac arrest

## Introduction

Traumatic Cardiac Arrest (TCA) precedes death in patients with traumatic injury. Although often associated with unsurvivable injuries, some cases are amenable to the correction of reversible causes. Injuries resulting in immediate TCA are only likely to be prevented by public health interventions. The causes of TCA are well documented and the pathologies that cause early death (e.g. airway obstruction, traumatic asphyxia, tension pneumothorax and cardiac tamponade) are more common in the pre-hospital population than the in-hospital population because many patients with these conditions do not survive to hospital. However, most immediate treatment interventions are the same regardless of where the patient presents. The range of interventions provided by pre-hospital services can be variable and depends on the skill set of providers on scene. This document is patient centred and necessary interventions are stated regardless of whether they can always be delivered in the pre-hospital phase of care. Some pre-hospital services may not be able to deliver all time critical interventions on scene and rapid transfer to hospital is then the only management option. Although hangings, electrocutions and drownings are often reported in studies of traumatic cardiac arrest they have different mechanisms and resuscitation considerations to traumatic cardiac arrest and are not covered in this position statement.

The aim of this document is to provide guidance for the pre-hospital management of TCA. The authors conducted a scoping review of literature relating to TCA published in English in the last 10 years (2014–2023). Databases searched included PubMed, EMBASE, UptoDate, BMJ Best Practice and the Cochrane Library. Reference lists of relevant publications were also searched. The searches are detailed in Appendix 1. The Faculty of Pre-hospital Care (FPHC) published a consensus statement on this topic in 2018 [1] and this document is an update informed by the most recent research available on the topic. There exist no high-quality comparative randomised controlled trials to inform us on the optimal treatment of pre-hospital TCA. Evidence for this document came largely from retrospectively collected observational data which is mostly level 3 evidence [2] and recommendations are therefore best practice statements based on expert opinion and evidence from in-hospital practice as well as the available pre-hospital evidence. Most relevant pre-hospital data was collected for, or extracted from, trauma registries. Trauma registry data is not representative of pre-hospital cases since it usually only includes patients who survive to hospital and is therefore subject to survival bias. Authors: All of the authors are clinically active in pre-hospital care. In addition they have in-hospital backgrounds in anaesthesia/intensive care medicine, trauma surgery and emergency medicine. The authors were nominated by the Faculty of Pre-hospital Care to conduct this project. The draft document was approved by the Executive group of the Faculty of Pre-hospital Care. Comments and changes were agreed and included in the final version.

Definition of TCA: Definitions of TCA vary in both guidelines and published TCA research. For this guideline the definition of TCA is an unresponsive pulseless patient following trauma.

### The challenges of TCA in the pre-hospital setting

The epidemiology and management of TCA has changed significantly in the UK and elsewhere due to changing patterns of trauma injury and the increased availability of advanced trauma care on scene. Many regions have seen an increase in penetrating trauma and reductions in road traffic injuries and work-related accidents. In parallel with these changes the proportion of severely injured patients attended by pre-hospital teams with advanced skills, particularly doctor—practitioner teams, has become routine in many regions. These teams deliver a range of treatment interventions previously only available after arrival in hospital. However, these teams may not always be on scene quickly and patients in TCA (or peri-arrest) usually have only a short time period for effective treatment. Although patients transported from scene in cardiac arrest rarely survive, patients with return of spontaneous circulation (ROSC) or those in a hypovolaemic peri-arrest state may need rapid transfer to in-hospital surgical intervention. Additional challenges for pre-hospital TCA management are the effective leadership and functioning of a ‘flash team’—a team of pre-hospital providers consisting of medical, paramedical and non-medical responders who have often never met before.

**Recommendation: Pre-hospital teams must rapidly evaluate critically unwell trauma patients and consider what resources are available on scene. Reversible pathologies must be rapidly identified, addressed and, where possible, rapid transfer carried out to an appropriate hospital.**

**Fig. 1:**
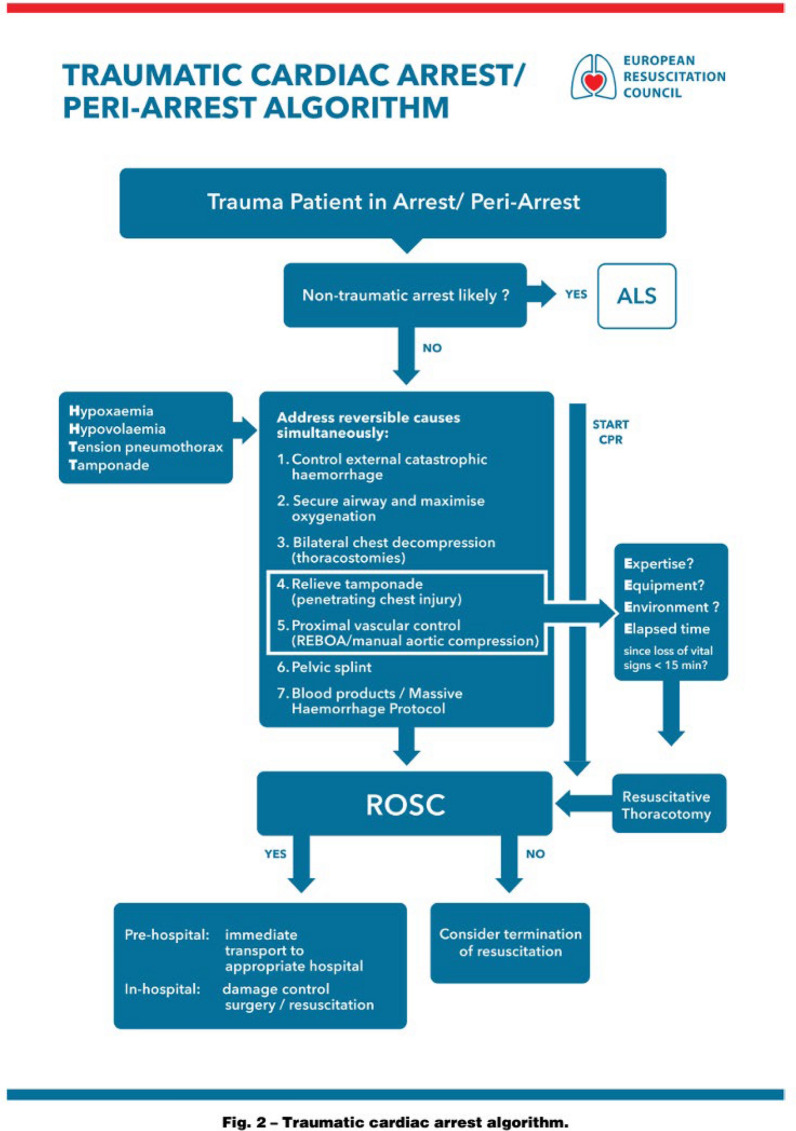
European Resuscitation Council TCA Algorithm 2021. Reproduced with permission of European Resuscitation Council

The mortality rate of TCA is high and there is little evidence that it has improved in recent years. A large systematic review and meta-analysis published in 2022 reported an overall mortality rate of 96.2% for TCA [3]. Reported mortality was 97.2% in studies including pre-hospital patients and 92.3% in studies excluding prehospital deaths. Of the few survivors, favourable neurological outcome rates were 35.8% in the studies which included prehospital deaths and 49.5% in studies excluding them. These findings were in keeping with a previous systematic review in 2012 [4]. Survival of patients transported in established cardiac arrest from scene to hospital is rare. Historically, TCA was sometimes considered unsurvivable and resuscitation efforts as futile [5, 6]. More recently this has been challenged. The European Resuscitation Council published its first TCA algorithm in 2010 which was updated in 2021 [7], based on an international consensus process [8]. The principles described in these guidelines are applicable to pre-hospital as well as in-hospital TCA (Fig. 1).

The underlying aetiology and physiology of TCA is different to medical cardiac arrest and this dictates the management priorities. TCA is often the result of a low cardiac output state, most commonly leading to pulseless-electrical activity [7]. It can be usefully divided into hypovolaemic cardiac arrest and TCA of other causes. TCA should be aggressively managed using a standardised approach that rapidly addresses reversible causes.

Survival is more likely if appropriate interventions can be delivered before the onset of cardiac arrest. These may include blood products or the treatment of airway obstruction or tension pneumothorax. Positive pressure ventilation may worsen haemodynamics in the trauma patient by impairing venous return to the heart. It may precipitate TCA in patients with tension pneumothorax, cardiac tamponade and severe hypovolaemia. In TCA, tension pneumothorax must be treated with thoracostomy before or immediately after intubation.

**Recommendation: Positive pressure ventilation can precipitate TCA in patients with hypovolaemia, cardiac tamponade or tension pneumothorax. These pathologies must be treated immediately if deterioration occurs.**

## Reversible causes of TCA

The management of pre-hospital TCA and peri-arrest should focus on stopping and replacing blood loss and addressing other reversible causes [8] **HOTT** (**H**ypovolaemia, **O**xygenation (hypoxia), **T**ension pneumothorax and cardiac **T**amponade) is a helpful mnemonic for remembering the key reversible causes of TCA [9]. TCA may be the result of any one or a combination of these processes [10]. It is often difficult to rapidly establish which factor is causative and for this reason a standardised approach to TCA addressing all possible reversible causes simultaneously is recommended.

**Recommendation: Addressing reversible factors in TCA is time critical. Use a standardised approach to address reversible factors simultaneously.**

TCA algorithms prioritise the immediate treatment of potentially reversible causes of arrest over the delivery of chest compressions. These interventions can and should be carried out quickly. The pre-hospital interventions available depend on the skills of the attending providers and can be variable. If teams are not proficient in the skills needed to perform the required interventions then follow standard ALS algorithms with urgent transfer and/or consideration of termination of resuscitation.

Some recent studies question the value of chest compressions in TCA [11], citing worse outcomes in animal studies and no-survival benefit in pre-hospital chest compressions compared no chest-compressions in TCA patients transferred to hospital for thoracotomy [12]. Chest compressions have a role in TCA, particularly in the minority of patients who are normovolaemic, but should not delay or compromise identification and treatment of reversible causes. In pre-hospital TCA the number of simultaneous interventions that can be delivered may depend on the number and skills of attending providers.

**Recommendation: Addressing the reversible causes of TCA has greater priority than chest compressions. Chest compressions are carried out with in normovolaemic TCA or where a medical cardiac arrest has preceded the traumatic episode.**

It is important to consider whether a medical cause has led to trauma and arrest (e.g. where a medical emergency leads to a fall or vehicle accident). If the cardiac arrest is the result of a medical cause rather than trauma, standard ALS protocols should be followed.

**Recommendation: If medical cardiac arrest is suspected deliver standard ALS interventions.**

## Hypovolaemia

Hypovolaemia in trauma is almost always due to haemorrhage. Hypovolaemia leads to a reduction in cardiac output and cardiac arrest results from inadequate coronary perfusion. Haemorrhage is the most common cause of TCA [7]. The priority in hypovolaemic TCA is to stop the bleeding, replace lost blood volume and achieve definitive haemorrhage control. Temporary haemorrhage control until in-hospital surgical intervention can be lifesaving.

External haemorrhage in the peri-arrest patient should be addressed via the haemostatic ladder (direct pressure, haemostatic agents, pressure dressings and tourniquets) to prevent arrest [13]. During cardiac arrest, blood flow is minimal and sources of external haemorrhage may not be obvious. Application of a pelvic binder or a tourniquet onto a bleeding extremity can prevent further blood loss if ROSC is achieved.

**Recommendation: Rapidly control external haemorrhage.**

Resuscitative emergency thoracotomy for proximal vascular control is advocated by the ERC as a last resort in TCA where expertise, equipment and environment allow and where time after arrest is less than 15 min [7]. Aortic occlusion can stop distal haemorrhage and improve blood flow to vital organs proximal to the occlusion (i.e. the heart and brain) [14]. Current evidence shows that those with penetrating chest injury have the best outcomes from thoracotomy. Survival after emergency room thoracotomy for penetrating or blunt trauma has been reported to be 8.5% [15]. A systematic review including 1,369 patients who underwent an ED thoracotomy following blunt trauma found that only 21 (1.5%) survived with a good neurological outcome [16]. Patients arriving in hospital in established cardiac arrest rarely survive and this, combined with the recommendation that thoracotomy should be delivered rapidly after arrest make pre-hospital thoracotomy, where possible, an appropriate intervention. The largest published series to date demonstrates better survival for patients with cardiac tamponade than those with exsanguination [17]. However, pre-hospital providers will often be unaware of what the underlying pathology is when commencing thoracotomy.

**Recommendation: Resuscitative thoracotomy is an established pre-hospital intervention in some pre-hospital services with doctor-practitioner teams. Where trained providers are available it is conducted as soon as possible after cardiac arrest. It is primarily indicated in penetrating chest trauma.**

The role of Resuscitative Endovascular Balloon Occlusion of the Aorta (REBOA) during TCA remains undetermined [18]. There is some evidence that REBOA can improve blood pressure and may increase ROSC rates and survival to discharge in TCA [19, 20]. However, the recent UK-REBOA trial examining the use of REBOA in non-compressible torso trauma was terminated early because of harm and the procedure was associated with increase mortality, increased deaths due to bleeding at 3 h and 90 days and delayed time to definitive haemorrhage control [21]. Further research may identify a cohort of patients who benefit from the intervention when performed by experienced physicians within a well-set up system that can expeditiously deliver the patient to definitive care. This intervention is likely to remain experimental for the immediate future [22].

**Recommendation: The evidence for benefit of REBOA and other aortic occlusion techniques are not yet established in advanced pre-hospital practice. These techniques should only be carried out as part of research studies or subject to specific patient indications and local protocols in services with comprehensive training and governance. These techniques must not delay definitive haemorrhage control.**

Blood and blood products are increasingly available in pre-hospital trauma resuscitation [23]. There is considerable heterogeneity in treatment protocols and products used by different services. Where correction of hypovolaemia is attempted blood products, in common with in-hospital trauma practice, are preferred over other fluids [24, 25]. Blood is a valuable resource and should be used when rapid clinical assessment suggests that successful resuscitation is a possibility. This is unlikely to be in all cases of TCA. Pre-hospital crystalloid solutions are not used to treat hypovolaemia leading to TCA unless blood products are not available.

**Recommendation: Blood and blood products are used early in resuscitation to manage hypovolaemia wherever possible. Local protocols currently determine the types of blood product used. Crystalloid solutions are not recommended unless cardiac arrest is imminent and blood products are not available.**

## Hypoxaemia

Optimisation of oxygenation in trauma patients is important for both immediate survival and for prevention of secondary injury. Hypoxaemia can result from airway obstruction, reduced level of conscious, ventilatory failure or traumatic asphyxia. All TCA patients should immediately be given high flow supplemental oxygen. Peripheral oxygen saturation readings are of limited use in the immediate management of the TCA patient but a useful confirmation of perfusion and oxygenation after ROSC has been achieved.

A patent airway is required to optimise oxygenation. Choice of airway management will depend on the skills and equipment available to the pre-hospital team. Management of the compromised airway may include more basic techniques such as bag-mask ventilation with supplemental oxygenation. More advanced management techniques include second generation supraglottic devices and tracheal intubation. Surgical cricothyroidotomy is reserved for the rare ‘can’t intubate, can’t ventilate’ scenario. This may be due to airway compromise or occasionally where access to the airway is not possible.

Intubated patients should be initially ventilated with 100% oxygen and continuous end-tidal CO2 monitoring is mandatory. Ventilation strategies should be lung protective with a tidal volume of 4–6 ml/kg targeting normocapnia and normoxia where ROSC is achieved.

**Recommendation: Correction of hypoxia is an immediate priority in TCA. Immediately administer high flow oxygen to patients in TCA. Airway patency and adequate ventilation is achieved with basic or more advanced airway interventions depending on the skills of attending providers.**

## Tension pneumothorax

In a tension pneumothorax, accumulating intrapleural air leads to lung collapse and the shift of mediastinal structures such as the heart and great vessels. Increased intrapleural pressure rises can collapse low pressure vessels (e.g. venae cavae) and obstruct venous return to the heart. This can ultimately lead to low flow states and the loss of cardiac output.

Large studies document an incidence of tension pneumothorax in TCA of between 6 [26] and 13% [27]. In suspected tension pneumothorax in TCA, needle decompression is no longer the first line treatment due to unacceptable failure and complication rates. When using a 5-cm catheter (e.g. 14G cannula) for anterior needle thoracostomy, based on computed tomography, the expected failure rate is 42.5% [28]. Complications include obstruction, kinking and displacement. Commercial large bore needle devices are available to reduce needle failure rates.

Bilateral thoracostomies to decompress the chest are indicated in TCA. They should be performed without pause for examination or diagnosis once TCA is established. Thoracostomies can be performed quickly and with a low complication rate [29, 30].

In critically ill ventilated patients thoracostomy has largely replaced the insertion of pre-hospital chest tubes. Tube insertion may delay arrival to definitive care and the drain can become kinked, displaced or blocked. After arrival at the receiving unit, a chest drain can be inserted in a sterile environment.

**Recommendation: In TCA decompress the chest with rapid bilateral thoracostomy or commercially available large bore needle decompression devices if thoracostomy is not available.**

## Cardiac tamponade

Filling of the pericardiac sac with blood can result in cardiac tamponade causing collapse of low pressure vessels and the atria. This limits ventricular filling during diastole, reduces cardiac output and can lead to TCA. Traumatic cardiac tamponade is usually due to penetrating injury but can rarely be due to blunt trauma and should be considered as a rare cause of TCA in patients with significant blunt chest trauma [31].

Patients with penetrating trauma causing a single ventricular wound and cardiac tamponade are likely to have better outcomes following surgical intervention than patients with blunt or more complex injury mechanisms [17, 31]. There is no role for pre-hospital needle pericardiocentesis during TCA due to penetrating cardiac trauma because clotted blood is usually present in the pericardial sac.

Resuscitative thoracotomy to relieve cardiac tamponade is advocated by the ERC in TCA where it has been less than 15 min since arrest and expertise, equipment and environment are available. Pre-hospital resuscitative thoracotomy is a procedure with a low rate of survival to discharge. Survival rates are higher when the intervention is carried out at, or minutes after, cardiac arrest [17]. The clamshell thoracotomy provides the best exposure and fastest control of thoracic injuries when compared to other approaches (e.g. a left anterolateral thoracotomy) [32].

Rapid transfer to the emergency department is the only option when a doctor trained in pre-hospital thoracotomy is not available to perform the procedure. Many pre-hospital emergency services in regions with low rates of penetrating trauma do not deliver this procedure.

**Recommendation: Cardiac tamponade causing TCA is rapidly fatal and must be immediately evacuated by thoracotomy on scene or, where not available, in the emergency department.**

## Ultrasound

The use of ultrasound is increasing in the pre-hospital environment. Potential uses in TCA include identifying pneumothoraces and cardiac tamponade and looking for cardiac activity as a prognostic tool [33].

Ultrasound must not delay or interrupt key interventions to treat the reversible causes of TCA and is usually not necessary in TCA after penetrating trauma. Only trained users should use intra-arrest ultrasound assessment.

**Recommendation: Ultrasound is a useful diagnostic pre-hospital tool but must not delay treatment of reversible pathologies.**

## Paediatric traumatic cardiac arrest

TCA in children is uncommon but accounts for a large proportion of paediatric out of hospital cardiac arrests (21%) [34]. A 2020 systematic review analysed 19 studies and reported a ROSC rate of 22.1%, event survival of 18.8% and survival to hospital discharge of 1.2% [35]. Children with a short period of TCA due to profound hypoxaemia or asphyxia have the best outcomes [36].

The immediate management of TCA in children is the same as for adults and focuses on treating the reversible causes (HOTT). Pre-hospital practitioners should be aware of specific paediatric considerations and ideally be experienced with caring for critically unwell children.

**Recommendation: The priorities for paediatric TCA are essentially the same as for adult TCA with particular emphasis on early hypoxaemia management. Paediatric provider skills must be regularly refreshed for this less common event.**

## Traumatic cardiac arrest in the obstetric patient

Trauma in pregnancy is a common cause of maternal death during pregnancy. However, data on trauma-related deaths is often not collected by registries that report on maternal death [37] so exact figures are not known. Maternal death is most commonly due to hypovolaemia secondary to haemorrhage while fetal death is often due to placental abruption.

During management of TCA in the pregnant patient, the patient should be in a supine position with manual uterine displacement to the left to reduce aorto-caval compression and improve venous return to the heart.

In obstetric patients more than 20 weeks gestation (or with a palpable uterus above the level of the umbilicus) a resuscitative hysterotomy may be performed to improve maternal outcome. The decision to perform a resuscitative hysterotomy should be made as soon as possible after TCA and the procedure carried out within a few minutes of the decision to operate [7, 38]. Although maternal survival has been reported to be much better when carried out within a few minutes of cardiac arrest survivors have been also been reported when commenced after this timeline [39]. This procedure is uncommonly reported in pre-hospital practice.

**Recommendation: Resuscitation of maternal TCA is be focussed on the same priorities as non-pregnant patients with uterine displacement and a focus on maternal survival.**

**Resuscitative hysterotomy can be considered after rapid assessment of injuries and timelines of cardiac arrest.**

## Termination of resuscitation

Several studies have attempted to identify factors which aid prognostication and identify patients who may have good outcomes following TCA. A recent study identified this topic as a priority research question in UK pre-hospital care [40]. The presence of cardiac motion on ultrasound or an initial shockable rhythm has been reported to be associated with increased survival [41]. TCA following penetrating injury is also associated with a higher survival and better outcomes [42]. Pulseless electrical activity, the most common rhythm seen in TCA, is not a positive prognostic factor [43]. There is some consensus that termination of resuscitation efforts should be considered if there is no ROSC after all reversible causes have been addressed or when there is no detectable cardiac activity on ultrasound.

The ERC TCA algorithm [7] (Fig. 1) recommends that resuscitation efforts are withheld when there are no signs of life in the preceding 15 min and where there is evidence of trauma incompatible with life. In major incidents and mass-casualty scenarios where scarce and valuable skills and resources may be required elsewhere it may be appropriate to withhold treatment in TCA. Several major incident publications and triage tools address this issue at national level.

## Supplementary Information

Additional file1 (DOCX 52 KB)

## Data Availability

No datasets were generated or analysed during the current study.
