# Supplementary material for: Pre-hospital management of traumatic cardiac arrest 2024 position statement: Faculty of Prehospital Care, Royal College of Surgeons of Edinburgh
Source: Scand J Trauma Resusc Emerg Med. 2024 Dec 31;32:139. doi: 10.1186/s13049-024-01304-z (PMC11686978; doi:10.1186/s13049-024-01304-z)
Supplement: Supplementary file 1 — Additional file1 (DOCX 52 KB) [file 13049_2024_1304_MOESM1_ESM.docx]

# **Appendix 1**

**Search Strategy**

10 year review: 2014-2023.

Databases searched: PubMed, UptoDate, BMJ Best Practice, Cochrane Library, Grey literature.

Keywords: Traumatic Cardiac Arrest, Pre-hospital, Cardiac Arrest in Trauma, Out of Hospital Cardiac Arrest

PubMed Search strategy: (("traumatic cardiac arrest"[Title/Abstract] OR "pre-hospital cardiac arrest"[Title/Abstract] OR "pediatric cardiac arrest"[Title/Abstract] OR "traumatic cardiac injury"[Title/Abstract] OR "traumatic cardiac tamponade"[Title/Abstract] AND ((y_10[Filter]) AND (english[Filter])) AND ((y_10[Filter]) AND (english[Filter]))) AND ((y_10[Filter]) AND (english[Filter]))) OR (((trauma[Title/Abstract] OR traumatic[Title/Abstract]) AND (((( "Out-of-Hospital Cardiac Arrest/complications"[Mesh] OR "Out-of-Hospital Cardiac Arrest/mortality"[Mesh] OR "Out-of-Hospital Cardiac Arrest/therapy"[Mesh] )) OR ( "Cardiopulmonary Resuscitation/adverse effects"[Mesh] OR "Cardiopulmonary Resuscitation/methods"[Mesh] OR "Cardiopulmonary Resuscitation/mortality"[Mesh] OR "Cardiopulmonary Resuscitation/trends"[Mesh] ) OR ( "Heart Arrest/therapy"[Mesh] ) AND ((y_10[Filter]) AND (english[Filter])) AND ((y_10[Filter]) AND (english[Filter]))) AND ((y_10[Filter]) AND (english[Filter])))) AND ((y_10[Filter]) AND (english[Filter]))

**174 results on Pubmed.** All reviewed.

# Contents:

1. Guidelines- 9
2. UptoDate topics- 8
3. Systematic reviews -10
4. Reviews- 34
5. Studies- 72
6. Case studies- 11
7. Animal and simulation studies- 7

Findings of literature search presented in reverse chronological order within each subheading.

## **Guidelines- 9 relevant**

# Major trauma: assessment and initial management NICE guideline [NG39] Published: 17 February 2016. <https://www.nice.org.uk/guidance/ng39> (Accessed 11/11/2024)

# Major trauma: service delivery NICE guideline [NG40] Published: 17 February 2016. <https://www.nice.org.uk/guidance/ng40> (Accessed 11/11/2024)

# Trauma Quality standard [QS166] Published: 29 March 2018. <https://www.nice.org.uk/guidance/qs166> (Accessed 11/11/2024)

- Royal College of Emergency Medicine: Best Practice Guideline Traumatic Cardiac Arrest in Adults 2019 <https://res.cloudinary.com/studiorepublic/> images/v1635411097/RCEM_ Traumatic_Cardiac_Arrest_Sept2019_FINAL/RCEM_Traumatic_Cardiac_Arrest_Sept2019_FINAL.pdf?_i=AAThe (Accessed 11/11/2024)
- The Royal College of Emergency Medicine: Position statement on Resuscitative Thoracotomy in Trauma Units. Position_Statement_on_Resuscitative_Thoracotomy-in_Trauma_Units_Apr_-2017.pdf (Accessed 11/11/2024)
- Perkins GD, Graesner JT, Semeraro F, Olasveengen T, Soar J, Lott C, Van de Voorde P, Madar J, Zideman D, Mentzelopoulos S, Bossaert L, Greif R, Monsieurs K, Svavarsdóttir H, Nolan JP; European Resuscitation Council Guideline Collaborators. European Resuscitation Council Guidelines 2021: Executive summary. Resuscitation. 2021 Apr;161:1-60.European
- Battaloglu, E; Porter, K Management of pregnancy and obstetric complications in prehospital trauma care: faculty of prehospital care consensus guidelines
  *Emergency Medicine Journal* Vol. 34 Issue 5, pp. 318–325, 2017.
- EMJ: Battaloglu E, Porter K. Management of pregnancy and obstetric complications in prehospital trauma care: prehospital resuscitative hysterotomy/perimortem caesarean section *Emergency Medicine Journal*2017;34:326-330

## **UptoDate Topics: 8 relevant**

- [Initial Management of Trauma in Adults](https://www.uptodate.com/contents/initial-management-of-trauma-in-adults?search=prehospital%20trauma%20cardiac%20arrest&topicRef=13838&source=see_link) (updated February 2023)
  - (The Primary Evaluation and Management section of this topic provides a review of current practice.)
- [Initial evaluation and management of blunt thoracic trauma in adults](https://www.uptodate.com/contents/initial-evaluation-and-management-of-blunt-thoracic-trauma-in-adults?search=traumatic%20cardiac%20arrest&source=search_result&selectedTitle=1~150&usage_type=default&display_rank=1) (updated March 2023)
- [Initial management of moderate to severe hemorrhage in the adult trauma patient](https://www.uptodate.com/contents/initial-management-of-moderate-to-severe-hemorrhage-in-the-adult-trauma-patient?search=prehospital%20trauma%20cardiac%20arrest&topicRef=13854&source=see_link) (updated March 2023)
- [Cardiac tamponade](https://www.uptodate.com/contents/cardiac-tamponade?search=prehospital%20trauma%20cardiac%20arrest&topicRef=127887&source=see_link) (updated August 2022)
- [Approach to shock in the adult trauma patient](https://www.uptodate.com/contents/approach-to-shock-in-the-adult-trauma-patient?search=prehospital%20trauma%20cardiac%20arrest&topicRef=13854&source=see_link) (updated February 2023)
- [Trauma management: Approach to the unstable child](https://www.uptodate.com/contents/trauma-management-approach-to-the-unstable-child?search=prehospital%20trauma%20cardiac%20arrest&topicRef=127887&source=see_link) (updated May 2022)
- [Trauma management: Unique pediatric considerations](https://www.uptodate.com/contents/trauma-management-unique-pediatric-considerations?search=prehospital%20trauma%20cardiac%20arrest&topicRef=13854&source=see_link) (updated June 2022)
- [Pediatric considerations in prehospital care](https://www.uptodate.com/contents/pediatric-considerations-in-prehospital-care?search=pediatric%20traumatic%20cardiac%20arrest&source=search_result&selectedTitle=2~150&usage_type=default&display_rank=2) (updated Sept 2022)

## **Systematic Reviews: 10 relevant**

- **Traumatic cardiac arrest**:
  - Vianen, Niek Johannes; Van Lieshout, Esther Maria Maartje; Maissan, Iscander Michael; Bramer, Wichor Matthijs; Hartog, Dennis Den; Verhofstad, Michael Herman Jacob; Van Vledder, Mark Gerrit. Prehospital traumatic cardiac arrest: a systematic review and meta-analysis. European Journal of Trauma and Emergency Surgery 2022 48 (4)3357–3372.
- **Airways**
  - Carney, N., Totten, A. M., Cheney, T., Jungbauer, R., Neth, M. R., Weeks, C., … Daya, M. (2021). Prehospital Airway Management: A Systematic Review. *Prehospital Emergency Care*, *26*(5), 716–727. Prehospital Airway Management: A Systematic Review. 2022. Prehospital Emergency Care.
- **Tension pneumothorax**
  - Robitaille-Fortin M, Norman S, Archer T, Mercier E. Prehospital Decompression of Pneumothorax: A Systematic Review of Recent Evidence. *Prehospital and Disaster Medicine*. 2021;36(4):450-459.
- **Hypovolaemia**
  - Hughes, M., Perkins, Z. Outcomes following resuscitative thoracotomy for abdominal exsanguination, a systematic review. *Scand J Trauma Resusc Emerg Med* **28**, 9 (2020).
- **Resuscitative thoracotomy**
  - Wang, M., Lu, X., Gong, P. *et al.* Open-chest cardiopulmonary resuscitation versus closed-chest cardiopulmonary resuscitation in patients with cardiac arrest: a systematic review and meta-analysis. *Scand J Trauma Resusc Emerg Med* **27**, 116 (2019).
  - Slessor D, Hunter S. To Be Blunt: Are We Wasting Our Time? Emergency Department Thoracotomy Following Blunt Trauma: A Systematic Review and Meta-Analysis, Annals of Emergency Medicine 2015; 65(3) 297-307.
- **Paediatrics**
  - Amagasa, Shunsuke; Utsumi, Shu; Moriwaki, Taro; Yasuda, Hideto; Kashiura, Masahiro; Uematsu, Satoko; Kubota, Mitsuru. Advanced airway management for pediatric out-of-hospital cardiac arrest: A systematic review and network meta-analysis. 2023. American Journal of Emergency Medicine. Vol. 68, pp. 161–169, 2023.
  - Alqudah, Zainab; Nehme, Ziad; Alrawashdeh, Ahmad; Williams, Brett; Oteir, Alaa; Smith, Karen Paediatric traumatic out-of-hospital cardiac arrest: A systematic review and meta-analysis *Resuscitation* 2020: 149,(65–73).
- **Ultrasound**
  - Lalande, Elizabeth; Burwash-Brennan, Talia; Burns, Katharine; Harris, Tim; Thomas, Stephen; Woo, Michael Y.; Atkinson, Paul. Is point-of-care ultrasound a reliable predictor of outcome during traumatic cardiac arrest? A systematic review and meta-analysis from the SHoC investigators. *Resuscitation*. 2021;167,(128–136).
- **Extras**
  - Manning, Sara. The Crashing Obese Patient *Emergency Medicine Clinics of North America*. Vol. 38 Issue 4, pp. 857–869, 2020.

## **Reviews- 34 relevant**

- **Traumatic cardiac arrest**
  - Breeding, Tessa; Martinez, Brian; Katz, Joshua; Kim, Jason; Havron, Will; Hoops, Heather; Elkbuli, Adel CAB versus ABC approach for resuscitation of patients following traumatic injury: Toward improving patient safety and survival
    *The American Journal of Emergency Medicine* Vol. 68, pp. 28–32, 2023.
  - Lewis, Jack; Perkins, Gavin D.Traumatic cardiac arrest *Current Opinion in Critical Care*
    Vol. 29 Issue 3, pp. 162–167, 2023.
  - Ramage, Lisa; McLachlan, Sarah; Williams, Kristian Determining the top research priorities in UK prehospital critical care: a modified Delphi study *Emergency Medicine Journal*. Vol. 40 Issue 4, pp. 271–276, 2023.
  - Barnard, Edward B.G.; Cesareo, Eric Traumatic cardiac arrest
    *Anaesthesia Critical Care & Pain Medicine* Vol. 41 Issue 3, p. 101077, 2022
  - Ordoobadi, Alexander J.; Peters, Gregory A.; MacAllister, Sean; Anderson, Geoffrey A.; Panchal, Ashish R.; Cash, Rebecca E. Prehospital care for traumatic cardiac arrest in the US: A cross-sectional analysis and call for a national guideline
    *Resuscitation.* Vol. 179, pp. 97–104, 2022.
  - Teeter, William; Haase, Daniel Updates in Traumatic Cardiac Arrest
    *Emergency Medicine Clinics of North America.* Vol. 38 Issue 4, pp. 891–901, 2020.
  - Wageningen, Bas van; Peters, Joost H.Suggestions to improve the traumatic cardiac arrest guidelines based on practical prehospital experience
    *Resuscitation* Vol. 164, p. 160, 2021.
  - Chinn M, Colella MR. Trauma Resuscitation: An evidence-based review of prehospital traumatic cardiac arrest. JEMS. 2017 Apr;42(4):26-32. PMID: 29220128.
  - Harris, Tim; Masud, Syed; Lamond, Anna; Abu-Habsa, Mamoun Traumatic cardiac arrest. *European Journal of Emergency Medicine* Vol. 22 Issue 2, pp. 72–78, 2015
  - Smith, Jason E; Rickard, Annette; Wise, David Traumatic cardiac arrest
    *Journal of the Royal Society of Medicine*. Vol. 108 Issue 1, pp. 11–16, 2015.
  - Lockey, David J.; Lyon, Richard M.; Davies, Gareth E. Development of a simple algorithm to guide the effective management of traumatic cardiac arrest *Resuscitation* Vol. 84 Issue 6, pp. 738–742, 2013.
- **Airway**
  - Aziz, Shadman; Foster, Elizabeth; Lockey, David J; Christian, Michael D Emergency scalpel cricothyroidotomy use in a prehospital trauma service: a 20-year review
    Emergency Medicine Journal. Vol. 38 Issue 5, pp. 349–354, 2021
- **Hypovolaemia**
  - Slot, S. A. S.; van Oostendorp, S. E.; Schoonmade, L. J.; Geeraedts, L. M. G.The role of REBOA in patients in traumatic cardiac arrest subsequent to hemorrhagic shock: a scoping review. European Journal of Trauma and Emergency Surgery .Vol. 49 Issue 2, pp. 693–707, 2023.
  - Hauswald, Mark; Kerr, Nancy L.External Aortic Compression in Noncompressible Truncal Hemorrhage and Traumatic Cardiac Arrest: A Scoping Review
    Annals of Emergency Medicine Vol. 80 Issue 2, pp. 175–176, 2022.
  - Aoki, Makoto; Abe, Toshikazu. Traumatic Cardiac Arrest: Scoping Review of Utilization of Resuscitative Endovascular Balloon Occlusion of the Aorta
    Frontiers in Medicine. Vol. 9, 2023.
  - ter Avest, E.; Carenzo, L.; Lendrum, R. A.; Christian, M. D.; Lyon, R. M.; Coniglio, C.; Rehn, M.; Lockey, D. J.; Perkins, Z. B. Advanced interventions in the pre-hospital resuscitation of patients with non-compressible haemorrhage after penetrating injuries. Critical Care Vol. 26 Issue 1, p. 184, 2022.
  - Iida, Atsuyoshi; Naito, Hiromichi; Nojima, Tsuyoshi; Yumoto, Tetsuya; Yamada, Taihei; Fujisaki, Noritomo; Nakao, Atsunori; Mikane, Takeshi State‐of‐the‐art methods for the treatment of severe hemorrhagic trauma: selective aortic arch perfusion and emergency preservation and resuscitation—what is next? Acute Medicine & Surgery Vol. 8 Issue 1, 2021.
  - Madurska, Marta J.; Ross, James D.; Scalea, Thomas M.; Morrison, Jonathan J.
    State-of-the-Art Review—Endovascular Resuscitation. Shock Vol. 55 Issue 3, pp. 288–300, 2021.
- **Tension pneumothorax**
  - Jodie, Pritchard; Kerstin, Hogg BET1: Pre-hospital finger thoracostomy in patients with traumatic cardiac arrest Emergency Medicine Journal Vol. 34 Issue 6, pp. 417.2–418, 2017.
- **Resuscitative thoracotomy**
  - Almond, Phillip; Morton, Sarah; OMeara, Matthew; Durge, Neal
    A 6-year case series of resuscitative thoracotomies performed by a helicopter emergency medical service in a mixed urban and rural area with a comparison of blunt versus penetrating trauma. *Scandinavian Journal of Trauma, Resuscitation and Emergency Medicine*. Vol. 30 Issue 1, p. 8, 2022.
  - Aseni, Paolo; Rizzetto, Francesco; Grande, Antonino M.; Bini, Roberto; Sammartano, Fabrizio; Vezzulli, Federico; Vertemati, Maurizio Emergency Department Resuscitative Thoracotomy: Indications, surgical procedure and outcome. A narrative review*The American Journal of Surgery* Vol. 221 Issue 5, pp. 1082–1092, 2021.
  - van Waes, Oscar J.F.; Leemeyer, Anna-Marie R.; Kooij, Fabian O.; Hoogerwerf, Nico; van Vledder, Mark G.Evaluation of out of hospital thoracotomy for cardiac arrest after penetrating thoracic trauma; Three years after our first report
    *Injury*. Vol. 50 Issue 11, pp. 2136–2137, 2019.
  - Fairfax, Lindsay M.; Hsee, Li; Civil, Ian D. Resuscitative Thoracotomy in Penetrating Trauma *World Journal of Surgery* Vol. 39 Issue 6, pp. 1343–1351, 2015.
- **Paediatrics**
  - Emigh, Brent; Grigorian, Areg; Dilday, Joshua; Condon, Freeman; Nahmias, Jeffry; Schellenberg, Morgan; Martin, Matthew; Matsushima, Kazuhide; Inaba, Kenji Risk factors and outcomes in pediatric blunt cardiac injuries. *Pediatric Surgery International*. Vol. 39 Issue 1, p. 195, 2023.
  - Stewart, Shai; Briggs, Kayla B.; Fraser, James A.; Svetanoff, Wendy Jo; Waddell, Valerie; Oyetunji, Tolulope A.Pre-hospital CPR after traumatic arrest: Outcomes at a level 1 pediatric trauma center. *Injury* Vol. 54 Issue 1, pp. 15–18, 2023.
  - VanDeWall, Audrey; Harris-Kober, Sarah; Farooqi, Ahmad; Kannikeswaran, Nirupama Peri-Intubation Arrest in High Risk vs. Standard Risk Pediatric Trauma Patients Undergoing Endotracheal Intubation. *The American Journal of Emergency Medicine*
    Vol. 67, pp. 79–83, 2023.
  - Tanner, Richard; Masterson, Siobhan; Galvin, Joseph; Wright, Peter; Hennelly, David; Murphy, Andrew; Bury, Gerard; O'Donnell, Cathal; Deasy, Conor Out-of-hospital cardiac arrests in the young population; a 6-year review of the Irish out-of-hospital cardiac arrest register. *Postgraduate Medical Journal* Vol. 97 Issue 1147, pp. 280–285, 2021.
  - Vassallo, James; Webster, Melanie; Barnard, Edward B G; Lyttle, Mark D; Smith, Jason E Epidemiology and aetiology of paediatric traumatic cardiac arrest in England and Wales. *Archives of Disease in Childhood*. Vol. 104 Issue 5, pp. 437–443, 2019.
  - Teague, Warwick J; Amarakone, Keith V; Quinn, Nuala. Rule of 4’s: Safe and effective pleural decompression and chest drain insertion in severely injured children
    *Emergency Medicine Australasia.* Vol. 31 Issue 4, pp. 683–687, 2019.
- **Withdrawal or termination**
  - Fierro, Nicole M.; Dhillon, Navpreet K.; Yong, Felix A.; Muniz, Tobias; Siletz, Anaar E.; Barmparas, Galinos; Ley, Eric J. No Resuscitative Thoracotomy? When to Stop Chest Compressions After Prehospital Traumatic Cardiac Arrest *American Surgeon*
    Vol. 88 Issue 10, pp. 2464–2469, 2022.
  - Currie, Victoria; Tagg, Andrew; Kanaris, Constantinos What information can we use to help determine futility in paediatric patients presenting in traumatic cardiac arrest? *Archives of Disease in Childhood* Vol. 107 Issue 7, pp. 695.1–697, 2022.
  - Khalifa, Andrew; Avraham, Jacob B.; Kramer, Kristina Z.; Bajani, Francesco; Fu, Chih Yuan; Pires-Menard, Alexandra; Kaminsky, Matthew; Bokhari, Faran. Surviving traumatic cardiac arrest: Identification of factors associated with survival
    *The American Journal of Emergency Medicine* Vol. 43, pp. 83–87, 2021.
  - Vassallo, James; Nutbeam, Tim; Rickard, Annette C; Lyttle, Mark D; Scholefield, Barney; Maconochie, Ian K; Smith, Jason E. Paediatric traumatic cardiac arrest: the development of an algorithm to guide recognition, management and decisions to terminate resuscitation *Emergency Medicine Journal*. Vol. 35 Issue 11, pp. 669–674, 2018.
  - Fallat, Mary E.; Cooper, Arthur; Salomone, Jeffrey; Mooney, David; Scherer, Tres; Wesson, David; Bulgar, et al. Withholding or Termination of Resuscitation in Pediatric Out-of-Hospital Traumatic Cardiopulmonary Arrest. *Pediatrics*. Vol. 133 Issue 4, pp. e1104–e1116, 2014.

## **Studies- 72 relevant**

- **Traumatic cardiac arrest**
  - Benhamed, Axel; Mercier, Eric; Freyssenge, Julie; Heidet, Mathieu; Gauss, Tobias; Canon, Valentine; Claustre, Clement; Tazarourte, Karim. Impact of the 2015 European guidelines for resuscitation on traumatic cardiac arrest outcomes and prehospital management: A French nationwide interrupted time-series analysis
    *Resuscitation*. Vol. 186, p. 109763, 2023.
  - Roche, Keelin F.; Quinn, Megan; Mannino, Elizabeth A.; Ventura, Liane; Brown, Cecelia; Lawson, Christy M.; Burns, Bracken J.A National Survey Assessing the Variability in the Management of Traumatic Cardiac Arrest
    *American Surgeon* Vol. 89 Issue 7, pp. 3125–3130, 2023.
  - Smida, Tanner; Price, Bradley S.; Scheidler, James; Crowe, Remle; Wilson, Alison; Bardes, James Stay and play or load and go? The association of on-scene advanced life support interventions with return of spontaneous circulation following traumatic cardiac arrest. *European Journal of Trauma and Emergency Surgery* Vol. 49 Issue 5, pp. 2165–2172, 2023.
  - Williamson, Frances; Lawton, Catherine F; Wullschleger, Martin. Outcomes in traumatic cardiac arrest patients who underwent advanced life support
    *Emergency Medicine Australasia* Vol. 35 Issue 2, pp. 205–212, 2023.
  - Prehospital predictors for return of spontaneous circulation in traumatic cardiac arrest Benhamed, Axel; Canon, Valentine; Mercier, Eric; Heidet, Matthieu; Gossiome, Amaury; Savary, Dominique; El Khoury, Carlos; Gueugniaud, Pierre-Yves; Hubert, Hervé; Tazarourte, Karim .*Journal of Trauma and Acute Care Surgery* Vol. 92 Issue 3, pp. 553–560, 2022.
  - Bujak, Kamil; Nadolny, Klaudiusz; Trzeciak, Przemysław; Gałązkowski, Robert; Ładny, Jerzy Robert; Gąsior, Mariusz Does the presence of physician-staffed emergency medical services improve the prognosis in out-of-hospital cardiac arrest? A propensity score matching analysis. *Polish Heart Journal* Vol. 80 Issue 6, pp. 685–692, 2022.
  - Doan, Tan N; Wilson, Daniel; Rashford, Stephen; Sims, Louise; Bosley, Emma Epidemiology, management and survival outcomes of adult out-of-hospital traumatic cardiac arrest due to blunt, penetrating or burn injury. *Emergency Medicine Journal*. Vol. 39 Issue 2, pp. 111–117, 2022.
  - Kitano, Shinnosuke; Fujimoto, Kenji; Suzuki, Kensuke; Harada, Satoshi; Narikawa, Kenji; Yamada, Marina; Nakazawa, Mayumi; Ogawa, Satoo; Yokota, Hiroyuki
    Evaluation of outcomes after EMS-witnessed traumatic out-of-hospital cardiac arrest caused by traffic collisions. *Resuscitation* Vol. 171, pp. 64–70, 2022.
  - Kuo, I-Ming; Chen, Yi-Fu; Chien, Chih-Ying; Hong, Yi-Wen; Kang, Shih-Ching; Fu, Chih-Yuan; Hsu, Chih-Po; Liao, Chien-Hung; Hsieh, Chi-Hsun A novel scoring system using easily assessible predictors of return of spontaneous circulation and mortality in traumatic out-of-hospital cardiac arrest patients: A retrospective cohort study
    *International Journal of Surgery*. Vol. 104, p. 106731, 2022.
  - Ohlén, Daniel; Hedberg, Magnus; Martinsson, Paula; von Oelreich, Erik; Djärv, Therese; Jonsson Fagerlund, Malin. Characteristics and outcome of traumatic cardiac arrest at a level 1 trauma centre over 10 years in Sweden.*Scandinavian Journal of Trauma, Resuscitation and Emergency Medicine*. Vol. 30 Issue 1, p. 54, 2022.
  - Ohlén, Daniel; Hedberg, Magnus; Martinsson, Paula; von Oelreich, Erik; Djärv, Therese; Jonsson Fagerlund, Malin Characteristics and outcome of traumatic cardiac arrest at a level 1 trauma centre over 10 years in Sweden. *Scandinavian Journal of Trauma, Resuscitation and Emergency Medicine* Vol. 30 Issue 1, p. 54, 2022.
  - Savary, Dominique; Morin, François; Douillet, Delphine; Drouet, Adrien; Ageron, François Xavier; Charvet, Romain; Carneiro, Bruno; Metton, Pierre; Fadel, Marc; Descatha, Alexis Impact of Specific Emergency Measures on Survival in Out-of-Hospital Traumatic Cardiac Arrest. *Prehospital and Disaster Medicine*. Vol. 37 Issue 1, pp. 51–56, 2022.
  - Seewald, Stephan; Wnent, Jan; Gräsner, Jan-Thorsten; Tjelmeland, Ingvild; Fischer, Matthias; Bohn, Andreas; Bouillon, Bertil; Maurer, Holger; Lefering, Rolf
    Survival after traumatic cardiac arrest is possible—a comparison of German patient-registries. *BMC Emergency Medicine*. Vol. 22 Issue 1, p. 158, 2022.
  - Tazarourte, Karim; Ageron, François-Xavier; Avondo, Aurélie; Barnard, Edward; Bobbia, Xavier; Cesareo, Eric; Chollet-Xemard, et al. Prehospital trauma flowcharts — Concise and visual cognitive aids for prehospital trauma management from the French Society of Emergency Medicine (SFMU) and the French Society of Anaesthesia and Intensive Care Medicine (SFAR) *Anaesthesia Critical Care & Pain Medicine*. Vol. 41 Issue 3, p. 101070, 2022.
  - Tisherman, Samuel A.Emergency preservation and resuscitation for cardiac arrest from trauma. *Annals of the New York Academy of Sciences*. Vol. 1509 Issue 1, pp. 5–11, 2022.
  - Alqudah, Zainab; Nehme, Ziad; Williams, Brett; Oteir, Alaa; Bernard, Stephen; Smith, Karen. Impact of a trauma-focused resuscitation protocol on survival outcomes after traumatic out-of-hospital cardiac arrest: An interrupted time series analysis
    *Resuscitation* Vol. 162, pp. 104–111, 2021
  - Alqudah, Zainab; Nehme, Ziad; Williams, Brett; Oteir, Alaa; Smith, Karen
    Survival outcomes in emergency medical services witnessed traumatic out-of-hospital cardiac arrest after the introduction of a trauma-based resuscitation protocol *Resuscitation* Vol. 168, pp. 65–74, 2021.
  - Savary, Dominique; Douillet, Delphine; Morin, François; Drouet, Adrien; Moumned, Thomas; Metton, Pierre; Carneiro, Bruno; Fadel, Marc; Descatha, Alexis Acting on the potentially reversible causes of traumatic cardiac arrest: Possible but not sufficient. *Resuscitation* Vol. 165, pp. 8–13, 2021.
  - Kim, Jae Guk; Lee, Juncheol; Choi, Hyun Young; Kim, Wonhee; Kim, Jihoon; Moon, Shinje; Shin, Hyungoo; Ahn, Chiwon; Cho, Youngsuk; Shin, Dong Geum; Lee, Yoonje
    Outcome analysis of traumatic out-of-hospital cardiac arrest patients according to the mechanism of injury. *Medicine (Wolters Kluwer)* Vol. 99 Issue 45, p. e23095, 2020.
  - Leemeyer, Anna-Marie R.; Van Lieshout, Esther M.M.; Bouwens, Maneka; Breeman, Wim; Verhofstad, Michael H.J.; Van Vledder, Mark G. Decision making in prehospital traumatic cardiac arrest; A qualitative study *Injury*. Vol. 51 Issue 5, pp. 1196–1202, 2020.
  - Aoki, Makoto; Abe, Toshikazu; Oshima, Kiyohiro Association of Prehospital Epinephrine Administration With Survival Among Patients With Traumatic Cardiac Arrest Caused By Traffic Collisions. *Scientific Reports*. Vol. 9 Issue 1, p. 9922, 2019.
  - Barnard, Ed B G; Sandbach, Daniel D; Nicholls, Tracy L; Wilson, Alastair W; Ercole, Ari Prehospital determinants of successful resuscitation after traumatic and non-traumatic out-of-hospital cardiac arrest. *Emergency Medicine Journal*. Vol. 36 Issue 6, pp. 333–339, 2019.
  - Yamamoto, Ryo; Suzuki, Masaru; Hayashida, Kei; Yoshizawa, Jo; Sakurai, Atsushi; Kitamura, Nobuya; Tagami, Takashi; Nakada, Taka-aki; Takeda, Munekazu; Sasaki, Junichi. Epinephrine during resuscitation of traumatic cardiac arrest and increased mortality: a post hoc analysis of prospective observational study.*Scandinavian Journal of Trauma, Resuscitation and Emergency Medicine*. Vol. 27 Issue 1, p. 74, 2019.
  - Barnard, Ed; Yates, David; Edwards, Antoinette; Fragoso-Iñiguez, Marisol; Jenks, Tom; Smith, Jason E. Epidemiology and aetiology of traumatic cardiac arrest in England and Wales — A retrospective database analysis. *Resuscitation*. Vol. 110, pp. 90–94, 2017.
  - Duchateau, François-Xavier; Hamada, Sophie; Raux, Mathieu; Gay, Matthieu; Mantz, Jean; Paugam Burtz, Catherine; Gauss, Tobias. Long-term prognosis after out-of-hospital resuscitation of cardiac arrest in trauma patients: prehospital trauma-associated cardiac arrest. *Emergency Medicine Journal*
    Vol. 34 Issue 1, pp. 34–38, 2017.
  - Lin, Chun-Yu; Tsai, Feng-Chun; Lee, Hsiu-An; Tseng, Yuan-His.Extracorporeal membrane oxygenation support in post-traumatic cardiopulmonary failure. *Medicine (Wolters Kluwer)* Vol. 96 Issue 6, p. e6067, 2017.
  - Fernandez AR. Is Resuscitation in Traumatic Cardiac Arrest Really Futile? EMS World. 2017 Mar;46(3):17-19. PMID: 29847029.
  - Lin, Chun-Yu; Tsai, Feng-Chun; Lee, Hsiu-An; Tseng, Yuan-His. Extracorporeal membrane oxygenation support in post-traumatic cardiopulmonary failure. *Medicine (Wolters Kluwer)* Vol. 96 Issue 6, p. e6067, 2017
  - Escott MEA. Fighting Futility. Traumatic cardiac arrest survivability is possible. JEMS. 2016 Jul;41(7):83.
  - Evans, Christopher C.D.; Petersen, Ashley; Meier, Eric N.; Buick, Jason E.; Schreiber, Martin; Kannas, Delores; Austin, Michael A. Prehospital traumatic cardiac arrest *Journal of Trauma and Acute Care Surgery*. Vol. 81 Issue 2, pp. 285–293, 2016.
  - Evans, Christopher C.D.; Petersen, Ashley; Meier, Eric N.; Buick, Jason E.; Schreiber, Martin; Kannas, Delores; Austin, Michael A. Prehospital traumatic cardiac arrest *Journal of Trauma and Acute Care Surgery*. Vol. 81 Issue 2, pp. 285–293, 2016.
  - Kleber, C.; Giesecke, M.T.; Lindner, T.; Haas, N.P.; Buschmann, C.T.
    Requirement for a structured algorithm in cardiac arrest following major trauma: Epidemiology, management errors, and preventability of traumatic deaths in Berlin *Resuscitation* Vol. 85 Issue 3, pp. 405–410, 2014.
- **Airways**
  - Nishimura, Takeshi; Suga, Masafumi; Nakao, Atsunori; Ishihara, Satoshi; Naito, Hiromichi Prehospital advanced airway management of emergency medical service‐witnessed traumatic out‐of‐hospital cardiac arrest patients: analysis of nationwide trauma registry.*Acute Medicine & Surgery* Vol. 9 Issue 1, 2022.
  - Le Bastard, Quentin; Rouzioux, Jade; Montassier, Emmanuel; Baert, Valentine; Recher, Morgan; Hubert, Hervé; Leteurtre, Stéphane; Javaudin, François Endotracheal intubation versus supraglottic procedure in paediatric out-of-hospital cardiac arrest: a registry-based study *Resuscitation*. Vol. 168, pp. 191–198, 2021.
- **Tension pneumothorax**
  - Peters, Joost; Ketelaars, Rein; van Wageningen, Bas; Biert, Jan; Hoogerwerf, Nico Prehospital thoracostomy in patients with traumatic circulatory arrest: results from a physician-staffed Helicopter Emergency Medical Service *European Journal of Emergency Medicine*. Vol. 24 Issue 2, pp. 96–100, 2017.
  - Escott ME, Gleisberg GR, Kimmel K, Karrer A, Cosper J, Monroe BJ. Simple thoracostomy. Moving beyong needle decompression in traumatic cardiac arrest. JEMS. 2014 Apr;39(4):26-32.
- **Resuscitative thoracotomy**
  - Stretch, Benjamin; Gomez, Denise Resuscitative thoracotomy in blunt traumatic cardiac arrest. *Scandinavian Journal of Trauma, Resuscitation and Emergency Medicine*. Vol. 30 Issue 1, p. 30, 2022.
  - Fitch, Jamie L.; Dieffenbaugher, Sean; McNutt, Michelle; Miller, C. Cody; Wainwright, D'Arcy J.; Villarreal, Joshua A.; Wilson, Chad T.; Todd, S. Rob
    Are We Out of the Woods Yet? The Aftermath of Resuscitative Thoracotomy
    *Journal of Surgical Research*.Vol. 245, pp. 593–599, 2020.
  - Tabiner, Nicholas Resuscitative thoracotomy for traumatic cardiac arrest: Clinical evidence and clinical governance. *Resuscitation* Vol. 139, p. 200, 2019.
  - Van Vledder, Mark G.; Van Waes, Oscar J.F.; Kooij, Fabian O.; Peters, Joost H.; Van Lieshout, Esther M.M.; Verhofstad, Michael H.J. Out of hospital thoracotomy for cardiac arrest after penetrating thoracic trauma *Injury*. Vol. 48 Issue 9, pp. 1865–1869, 2017.
  - Ottestad, William; Bredmose, Per B.; Berve, Per Olav; Stave, Halvard; Farstad, Gunnar; Wik, Lars; Sandberg, Mårten Prehospital thoracotomy for traumatic cardiac arrest. Vol. 136 Issue 23/24, pp. 1964–1965, 2016.
  - Pust, Gerd Daniel; Namias, Nicholas Resuscitative thoracotomy *International Journal of Surgery*.Vol. 33 Issue PB, pp. 202–208, 2016.
  - Mitchell, Thomas A.; Waldrep, Kevin B.; Sams, Valerie G.; Wallum, Timothy E.; Blackbourne, Lorne H.; White, Christopher E.An 8-Year Review of Operation Enduring Freedom and Operation Iraqi Freedom Resuscitative Thoracotomies
    *Military Medicine* Vol. 180 Issue 3S, pp. 33–36, 2015.
  - Chalkias, A.; Xanthos, T. Should prehospital resuscitative thoracotomy be incorporated in advanced life support after traumatic cardiac arrest?
    *European Journal of Trauma and Emergency Surgery* Vol. 40 Issue 3, pp. 395–397, 2014.
- **Hypovolaemia**
  - Gamberini, Lorenzo; Coniglio, Carlo; Lupi, Cristian; Tartaglione, Marco; Mazzoli, Carlo Alberto; Baldazzi, Marzia; Cecchi, Alessandra; Ferri, Enrico; Chiarini, Valentina; Semeraro, Federico; Gordini, Giovanni. Resuscitative endovascular occlusion of the aorta (REBOA) for refractory out of hospital cardiac arrest. An Utstein-based case series *Resuscitation* Vol. 165, pp. 161–169, 2021.
  - Theodorou, Christina M.; Trappey, A. Francois; Beyer, Carl A.; Yamashiro, Kaeli J.; Hirose, Shinjiro; Galante, Joseph M.; Beres, Alana L.; Stephenson, Jacob T. Quantifying the need for pediatric REBOA: A gap analysis *Journal of Pediatric Surgery*. Vol. 56 Issue 8, pp. 1395–1400, 2021.
  - Balian, Fay; Garner, Alan A.; Weatherall, Andrew; Lee, Anna First experience with the abdominal aortic and junctional tourniquet in prehospital traumatic cardiac arrest *Resuscitation* Vol. 156, pp. 210–214, 2020
  - Fitzgerald, Mark; Lendrum, Robbie; Bernard, Stephen; Moloney, John; Smit, De Villiers; Mathew, Joseph; Kim et al Feasibility study for implementation of resuscitative balloon occlusion of the aorta in peri‐arrest, exsanguinating trauma at an adult level 1 Australian trauma centre *Emergency Medicine Australasia*
    Vol. 32 Issue 1, pp. 127–134, 2020.
  - Hilbert-Carius, Peter; McGreevy, David T.; Abu-Zidan, Fikri M.; Hörer, Tal M.Pre-hospital CPR and early REBOA in trauma patients — results from the ABO Trauma Registry.*World Journal of Emergency Surgery* Vol. 15 Issue 1, p. 23, 2020.
  - McGreevy, David Thomas; Abu-Zidan, Fikri M.; Sadeghi, Mitra; Pirouzram, Artai; Toivola, Asko; Skoog, Per; Idoguchi, Koji; Kon, Yuri; Ishida, Tokiya; Matsumura, Yosuke; Matsumoto, Junichi; Reva et al Feasibility and Clinical Outcome of Reboa in Patients with Impending Traumatic Cardiac Arrest
    *Shock*, Vol. 54 Issue 2, pp. 218–223, 2020.
  - Yamamoto, Ryo; Suzuki, Masaru; Funabiki, Tomohiro; Nishida, Yusho; Maeshima, Katsuya; Sasaki, Junichi Resuscitative endovascular balloon occlusion of the aorta and traumatic out‐of‐hospital cardiac arrest: A nationwide study. *JACEP Open : Journal of the American College of Emergency Physicians Open*. Vol. 1 Issue 4, pp. 624–632, 2020.
  - Henry, Reynold; Matsushima, Kazuhide; Henry, Rachel N.; Wong, Victor; Warriner, Zachary; Strumwasser, Aaron; Foran, Christopher P.; Inaba, Kenji; Rasmussen, Todd E.; Demetriades, Demetrios Who Would Have Benefited from the Prehospital Use of Resuscitative Endovascular Balloon Occlusion of the Aorta (REBOA)? An Autopsy Study *Journal of the American College of Surgeons*. Vol. 229 Issue 4, pp. 383–388e1, 2019.
  - Rottenberg, Eric M.The need for abdominal only CPR in the treatment of hemorrhagic shock and trauma arrests *The American Journal of Emergency Medicine* Vol. 34 Issue 6, pp. 1156–1157, 2016
- **Paediatrics**
  - Lockhart-Bouron M, Baert V, Leteurtre S, Hubert H, Recher M. Association between out-of-hospital cardiac arrest and survival in paediatric traumatic population: results from the French national registry. Eur J Emerg Med. 2023 Jun 1;30(3):186-192.
  - Samuel N, Hoffmann Y, Rakedzon S, Lipsky AM, Raz A, Ben Lulu H, Bahouth H, Epstein D. Indications for prehospital intubation among severely injured children and the prevalence of significant traumatic brain injury among those intubated due to impaired level of consciousness. Eur J Trauma Emerg Surg. 2023 Jun;49(3):1217-1225.
  - El Tawil, Chady; LeBlanc, Pierre-Alexandre; Beno, Suzanne; Nemeth, Joe Traumatic cardiac arrest: unique considerations for the pediatric patient *Canadian Journal of Emergency Medicine* Vol. 24 Issue 4, pp. 457–458, 2022.
  - El Tawil, Chady; LeBlanc, Pierre-Alexandre; Beno, Suzanne; Nemeth, Joe
    Traumatic cardiac arrest: unique considerations for the pediatric patient
    *Canadian Journal of Emergency Medicine* Vol. 24 Issue 4, pp. 457–458, 2022.
  - Faulkner, Justin; Carballo, Christopher; Colosimo, Christina; Gratton, Austin; Mentzer, Caleb; Yon, James Traumatic Cardiac Arrest in Pediatric Patients: An Analysis of the National Trauma Database 2007-2016 *American Surgeon* Vol. 88 Issue 9, pp. 2252–2254, 2022.
  - Lanyi, Maria; Elmer, Jonathan; Guyette, Francis X.; Martin-Gill, Christian; Venkat, Arvind; Traynor, Owen; Walker, Heather; Seaman, Kristen; Kochanek, Patrick M.; Fink, Ericka L.Survival Rates After Pediatric Traumatic Out-of-Hospital Cardiac Arrest Suggest an Underappreciated Therapeutic Opportunity *Pediatric Emergency Care* Vol. 38 Issue 9, pp. 417–422, 2022.
  - Lelak, Karima A.; Arora, Rajan; Mowbray, Fabrice I.; Arkatkar BS, Anooj; Krouse, Carolyn; Cloutier, Dawn; Donoghue, Lydia; Sethuraman, Usha Cardiopulmonary Resuscitation and Epinephrine Use in Pediatric Traumatic Cardiac Arrest
    *American Surgeon* Vol. 89 Issue 6, pp. 2965–2968, 2023.
  - Ngatuvai, Micah; Rosander, Abigail; Elkbuli, Adel. Improving Outcomes and Resuscitation Strategies for Traumatic Cardiac Arrest in the Pediatric Population *American Surgeon* Vol. 90 Issue 1, pp. 166–167, 2024.
  - Gardner, Monique M.; Topjian, Alexis A. Improving outcomes from pediatric cardiac arrest: Should we be out for blood? *Resuscitation* Vol. 167, pp. 405–406, 2021.
  - Quinn, Nuala; Palmer, Cameron S; Bernard, Stephen; Noonan, Michael; Teague, Warwick J Thoracostomy in children with severe trauma: An overview of the paediatric experience in Victoria, Australia *Emergency Medicine Australasia*
    Vol. 32 Issue 1, pp. 117–126, 2020.
  - Alqudah, Zainab; Nehme, Ziad; Williams, Brett; Oteir, Alaa; Bernard, Stephen; Smith, Karen A descriptive analysis of the epidemiology and management of paediatric traumatic out-of-hospital cardiac arrest *Resuscitation* Vol.140,  pp. 127–134, 2019.
  - Hillman, Christopher M; Rickard, A; Rawlins, M; Smith, JE Paediatric traumatic cardiac arrest: data from the Joint Theatre Trauma Registry *BMJ Military Health* Vol. 162 Issue 4, pp. 276–279, 2016
  - Zwingmann, Jörn; Lefering, Rolf; Bayer, Jörg; Reising, Kilian; Kuminack, Kerstin; Südkamp, Norbert P.; Strohm, Peter C. Outcome and risk factors in children after traumatic cardiac arrest and successful resuscitation *Resuscitation*
    Vol. 96, pp. 59–65, 2015.
- **Withdrawing or termination**
  - Harris, Matthew I.; Crowe, Remle P.; Anders, Jennifer; D'Acunto, Salvatore; Adelgais, Kathleen M.; Fishe, Jennifer Applying a set of termination of resuscitation criteria to paediatric out-of-hospital cardiac arrest
    *Resuscitation* Vol. 169, pp. 175–181, 2021.
  - Naito, Hiromichi; Yumoto, Tetsuya; Yorifuji, Takashi; Nojima, Tsuyoshi; Yamamoto, Hirotsugu; Yamada, Taihei; Tsukahara, Kohei; Inaba, Mototaka; Nishimura, Takeshi; Uehara, Takenori; Nakao, Atsunori Association between emergency medical service transport time and survival in patients with traumatic cardiac arrest: a Nationwide retrospective observational study *BMC Emergency Medicine*
    Vol. 21 Issue 1, p. 104, 2021.
  - Shibahashi, Keita; Sugiyama, Kazuhiro; Hamabe, Yuichi Pediatric Out-of-Hospital Traumatic Cardiopulmonary Arrest After Traffic Accidents and Termination of Resuscitation *Annals of Emergency Medicine* Vol. 75 Issue 1, pp. 57–65, 2020.
  - Israr, S; Cook, AD; Chapple, KM; Jacobs, JV; McGeever, KP; Tiffany, BR; Schultz, SP; Petersen, SR; Weinberg, JA Pulseless electrical activity following traumatic cardiac arrest: Sign of life or death? *Injury* Vol. 50 Issue 9, pp. 1507–1510, 2019.
  - Norii, Tatsuya; Matsushima, Kazuhide; Miskimins, Richard J; Crandall, Cameron S
    Should we resuscitate elderly patients with blunt traumatic cardiac arrest? Analysis of National Trauma Registry Data in Japan *Emergency Medicine Journal*
    Vol. 36 Issue 11, pp. 670–677, 2019.
  - Beck, Ben; Bray, Janet E.; Cameron, Peter; Straney, Lahn; Andrew, Emily; Bernard, Stephen; Smith, Karen Resuscitation attempts and duration in traumatic out-of-hospital cardiac arrest *Resuscitation* Vol. 111, pp. 14–21, 2017.
  - Chiang, Wen-Chu; Huang, Yu-Sheng; Hsu, Shu-Hsien; Chang, Anna Marie; Ko, Patrick Chow-In; Wang, Hui-Chih; Yang, Chih-Wei; Hsieh, Ming-Ju; Huang, Edward Pei-Chuan; Chong, Kah-Meng; Sun, Jen-Tang; Chen, Shey-Ying; Ma, Matthew Huei-Ming. Performance of a simplified termination of resuscitation rule for adult traumatic cardiopulmonary arrest in the prehospital setting
    *Emergency Medicine Journal* Vol. 34 Issue 1, pp. 39–45, 2017.
  - Chien, Cheng-Yu; Su, Yi-Chia; Lin, Chi-Chun; Kuo, Chan-Wei; Lin, Shen-Che; Weng, Yi-Ming Is 15 minutes an appropriate resuscitation duration before termination of a traumatic cardiac arrest? A case-control study *The American Journal of Emergency Medicine* Vol. 34 Issue 3, pp. 505–509, 2016.
  - Rotering, Victoria Maria; Trepels-Kottek, Sonja; Heimann, Konrad; Brokmann, Jörg-Christian; Orlikowsky, Thorsten; Schoberer, Mark Adult “termination-of-resuscitation” (TOR)-criteria may not be suitable for children - a retrospective analysis *Scandinavian Journal of Trauma, Resuscitation and Emergency Medicine*
    Vol. 24 Issue 1, 2016.
  - Bawazeer, Manal; Al Alawyat, Hanaa; Zamakhshary, Mohammed Applicability of Adult Guidelines for Withholding or Terminating Resuscitation for Prehospital Traumatic Cardiopulmonary Arrest in Pediatrics *European Journal of Pediatric Surgery* Vol. 25 Issue 02, pp. 206–211, 2015.

## **Case studies- 11 relevant**

- de Assis, Viviana; Shields, Andrea D.; Johansson, Alaina; Shumbusho, Diane I.; York, Brian M.
  Resuscitation of traumatic maternal cardiac arrest: A case report and summary of recommendations from Obstetric Life Support™ *Trauma Case Reports* Vol. 44, p. 100800, 2023.
- Robinson, Aaron E.; Jones, Gregg A.; Nystrom, Paul C.; Stirling, Adam; Vanderbosch, Kelsey; Simpson, Nicholas S. Prehospital Pericardiocentesis Using a Pneumothorax Needle *Prehospital Emergency Care* Vol. 26 Issue 3, pp. 406–409, 2022.
- Lai, Jason; Kuttab, Hani; Newberry, Ryan; Stader, Michael; Cathers, Andrew Prehospital Ultrasound Use to Guide Resuscitative Thoracotomy in Blunt Traumatic Cardiac Arrest
  *Air Medical Journal* Vol. 41 Issue 5, pp. 494–497, 2022.
- Alageel M, Aldarwish NA, Alabbad FA, Alotaibi FM, Almania MN, Alshalawi SM. Refractory Ventricular Fibrillation in Traumatic Cardiac Arrest: A Case Report and Review of the Literature. Cureus. 2021 Nov 24;13(11):e19851
- Mapp, Julian G.; Manifold, Craig A.; Garcia, Alberto M.; Aguilar, Jason L.; Stringfellow, Michael L.; Winckler, Christopher J.Prehospital blunt traumatic arrest resuscitation augmented by whole blood: a case report *Transfusion* Vol. 60 Issue 5, pp. 1104–1107, 2020.
- Rogerson, T; Efstratiades, T; Von Oppell, U; Davies, G; Curtin, R Survival after pre-hospital emergency clamshell thoracotomy for blunt cardiac rupture *Injury* Vol. 51 Issue 1, pp. 122–123, 2020.
- Vreeswijk, Sebastiaan J.M.; Mommers, Lars P.W.; Bergmans, Dennis C.J.J.; van Wageningen, Bas
  Clamshell in a Heartbeat *Air Medical Journal* Vol. 39 Issue 6, pp. 509–511, 2020.
- Kjellemo, Hugo; Hansen, Andreas E.; Øines, Dennis A.; Nilsen, Thor O.; Wik, Lars Pediatric Cardiac Arrest Due to Trauma *Prehospital Emergency Care*. Vol. 20 Issue 3, pp. 425–431, 2016.
- McKenzie, Myles R; Parrish, Ernest W; Miles, Ethan A; Spradling, James C; Littlejohn, Lanny F; Quinlan, Mark D; Barbee, George A; King, David R A Case of Prehospital Traumatic Arrest in a US Special Operations Soldier: Care From Point of Injury to Full Recovery *J. Spec. Oper. Med.* Vol 16, Issue 3, p. 93, 2016.
- Gatti, Francesca; Spagnoli, Marco; Zerbi, Simone Maria; Colombo, Dario; Landriscina, Mario; Kette, Fulvio Out-of-Hospital Perimortem Cesarean Section as Resuscitative Hysterotomy in Maternal Posttraumatic Cardiac Arrest *Case Reports in Emergency Medicine* Vol. 2014, pp. 1–4, 2014.

## **Animal and simulation studies- 7 relevant**

- Anderson, Kenton L; Evans, Jacqueline C; Castaneda, Maria G; Boudreau, Susan M; Maddry, Joseph K; Morgan, Jeffrey D Effects of Left Ventricular Versus Traditional Chest Compressions in a Traumatic Pulseless Electrical Activity Model
  *Military Medicine* Vol. 187 Issue 3-4, pp. 351–359, 2022.
- Edwards, Joseph; Abdou, Hossam; Patel, Neerav; Lang, Eric; Richmond, Michael J.; Rasmussen, Todd E.; Scalea, Thomas M.; Morrison, Jonathan J. Open chest selective aortic arch perfusion vs open cardiac massage as a means of perfusion during in exsanguination cardiac arrest: a comparison of coronary hemodynamics in swine
  *European Journal of Trauma and Emergency Surgery* Vol. 48 Issue 3, pp. 2089–2096, 2022.
- Anderson, Kenton L.; Morgan, Jeffrey D.; Castaneda, Maria G.; Boudreau, Susan M.; Araña, Allyson A.; Kohn, Michael A.; Bebarta, Vikhyat S. The Effect of Chest Compression Location and Occlusion of the Aorta in a Traumatic Arrest Model *Journal of Surgical Research* Vol. 254, pp. 64–74, 2020
- Anderson, Kenton L.; Morgan, Jeffrey D.; Castaneda, Maria G.; Boudreau, Susan M.; Araña, Allyson A.; Kohn, Michael A.; Bebarta, Vikhyat S The Effect of Chest Compression Location and Occlusion of the Aorta in a Traumatic Arrest Model *Journal of Surgical Research*. Vol. 254, pp. 64–74, 2020.
- Hoops, Heather E.; Manning, James E.; Graham, Todd L.; McCully, Belinda H.; McCurdy, Shane L.; Ross, James D. Selective aortic arch perfusion with fresh whole blood or HBOC-201 reverses hemorrhage-induced traumatic cardiac arrest in a lethal model of noncompressible torso haemorrhage. *Journal of Trauma and Acute Care Surgery* Vol. 87 Issue 2, pp. 263–273, 2019.
- Douma, M.J.; O’Dochartaigh, D.; Brindley, P.G. Bi-manual proximal external aortic compression after major abdominal-pelvic trauma and during ambulance transfer: A simulation study *Injury* Vol. 48 Issue 1, pp. 26–31, 2017.
- Douma, Matthew J.; O’Dochartaigh, Domhnall; Brindley, Peter G. Optimization of indirect pressure in order to temporize life-threatening haemorrhage: A simulation study
  *Injury* Vol. 47 Issue 9, pp. 1903–1907, 2016
- Puchwein, Paul; Sommerauer, Florian; Clement, Hans G.; Matzi, Veronika; Tesch, Norbert P.; Hallmann, Barbara; Harris, Tim; Rigaud, Marcel Clamshell thoracotomy and open heart massage—A potential life-saving procedure can be taught to emergency physicians
  *Injury* Vol. 46 Issue 9, pp. 1738–1742, 2015.
